# Supplementary figures and images for: Environmental DNA metabarcoding reveals distinct fish assemblages supported by seagrass (Zostera marina and Zostera pacifica) beds in different geographic settings in Southern California
Source: PLoS One. 2023 Oct 5;18(10):e0286228. doi: 10.1371/journal.pone.0286228 (PMC10553302; doi:10.1371/journal.pone.0286228)

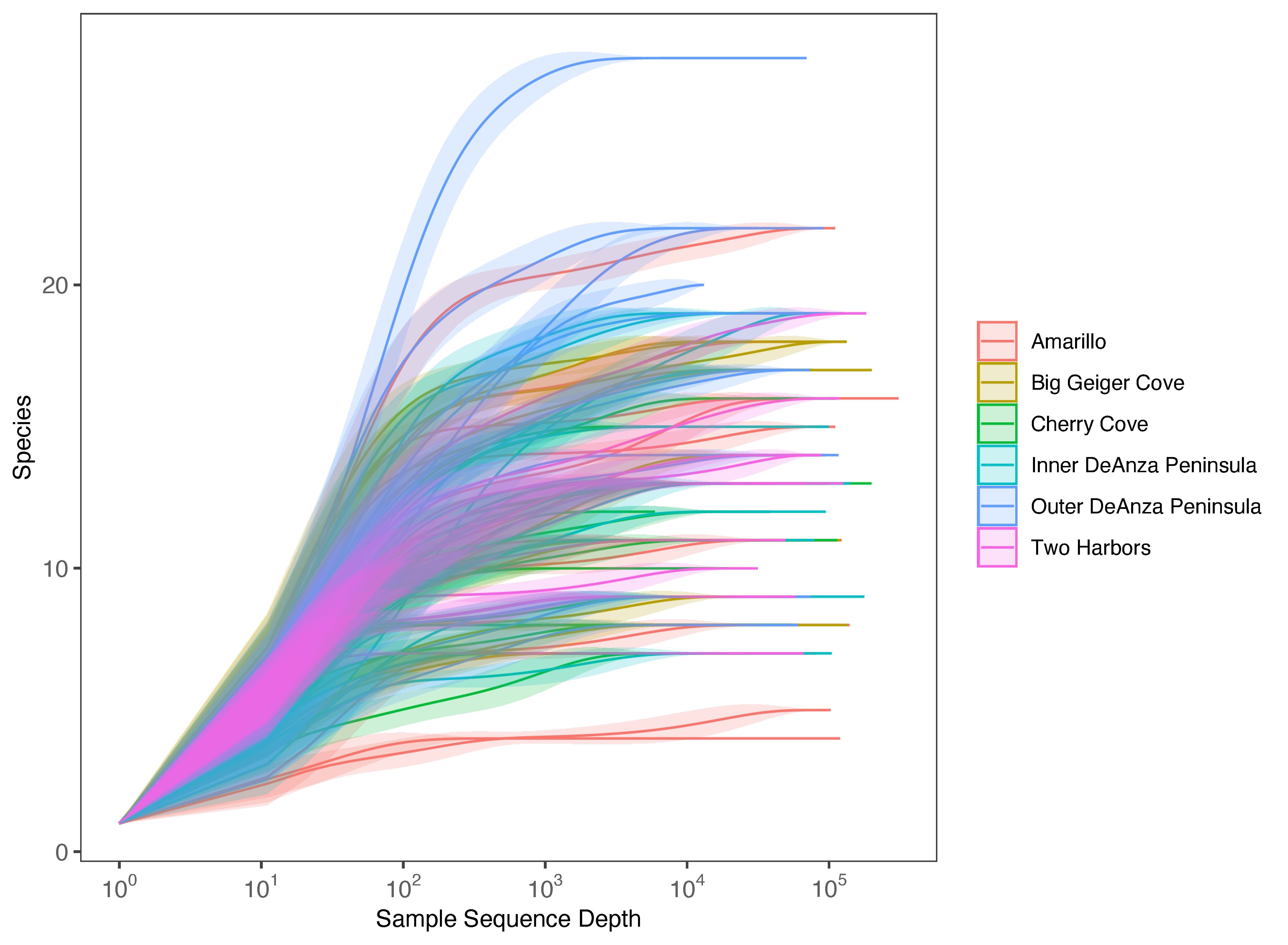

Supplement: S1 Fig — (TIF) [file pone.0286228.s001.tif]

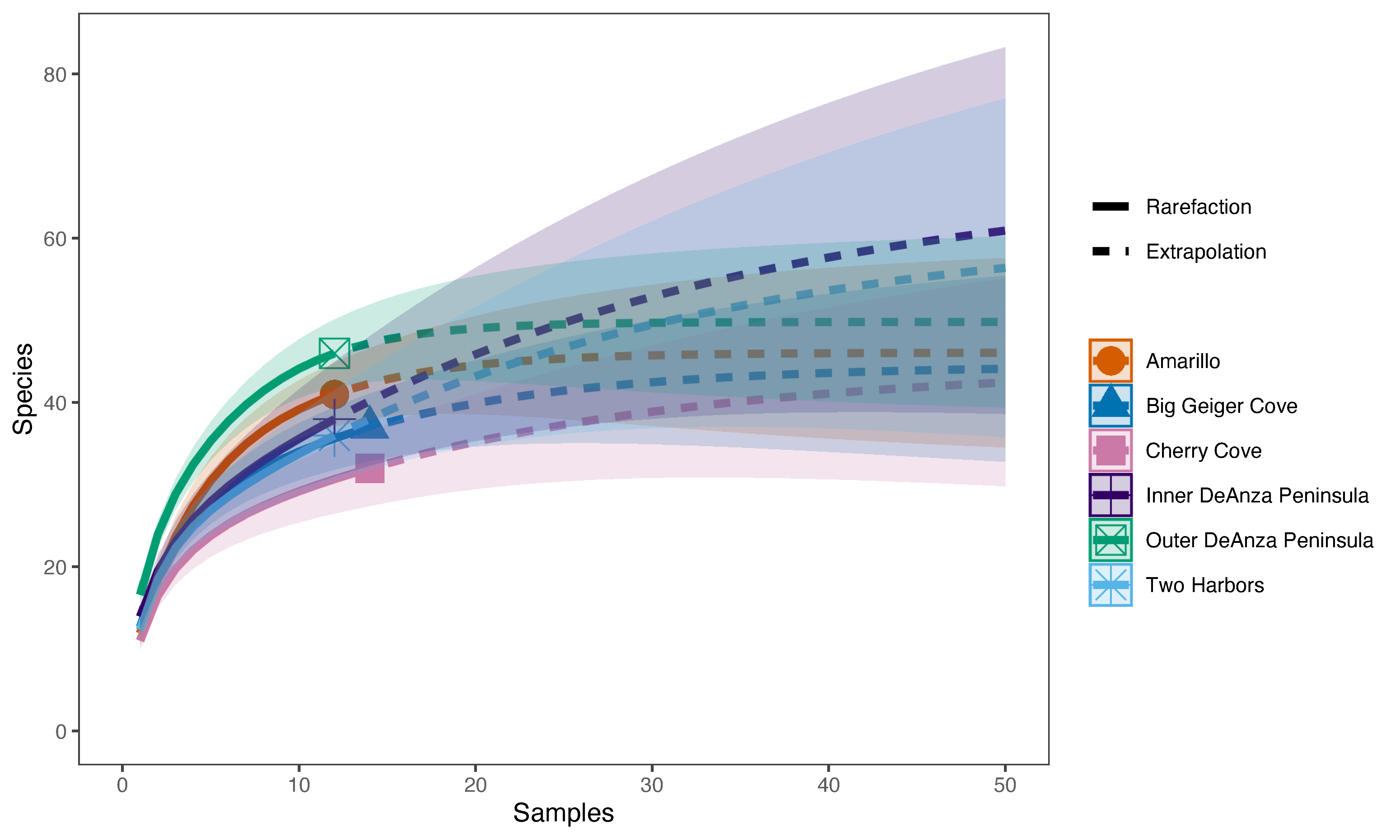

Supplement: S2 Fig — (TIF) [file pone.0286228.s002.tif]

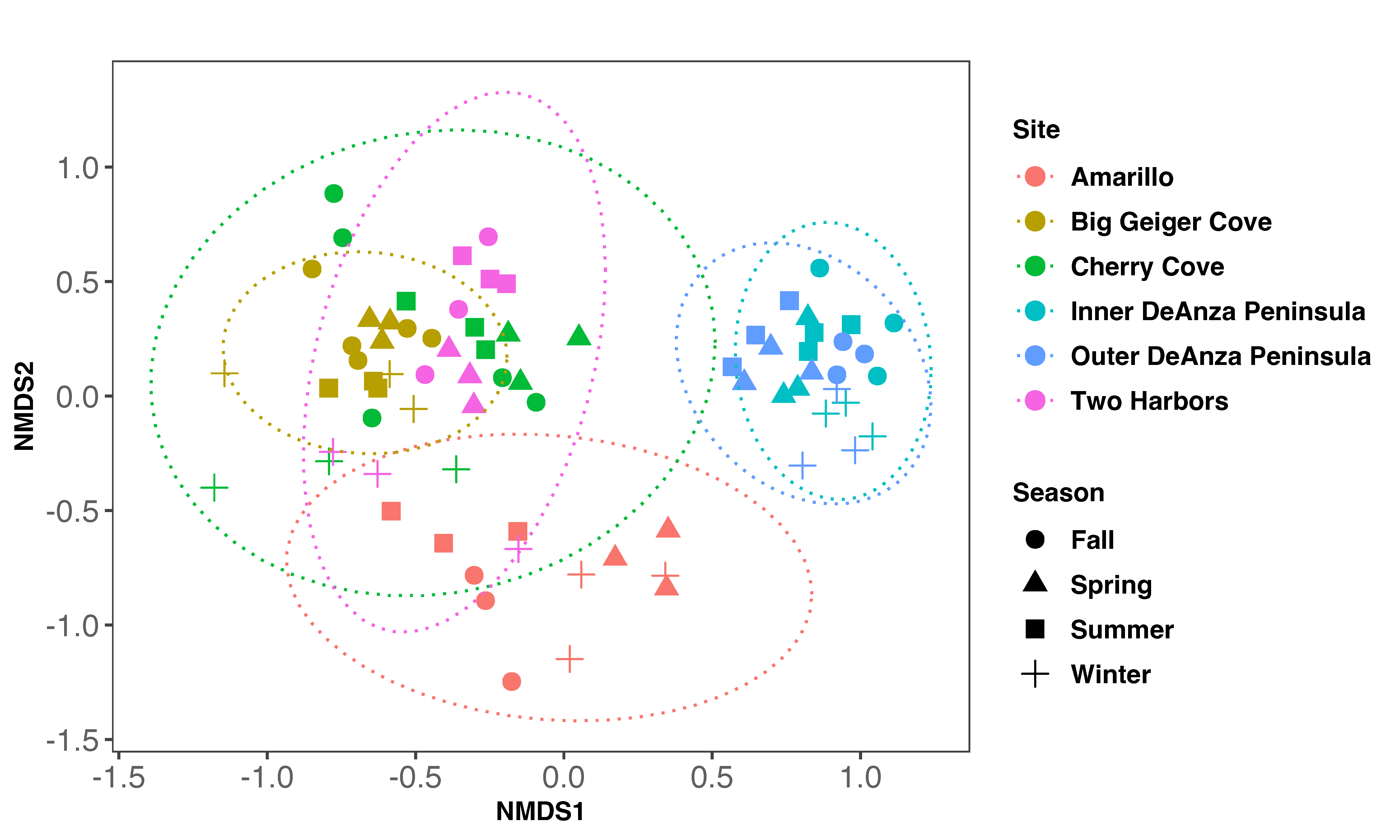

Supplement: S3 Fig — NMDS shows that community composition of seagrass beds is more strongly dependent on their geographical location, i.e. in an embayment (Inner and Outer Newport), open coast (Amarillo) or island (Big Geiger and Two Harbors), than the season of sampling. Colors indicate site and shapes indicate season. (TIF) [file pone.0286228.s003.tif]
