# Supplementary material for: Environmental DNA metabarcoding reveals distinct fish assemblages supported by seagrass (Zostera marina and Zostera pacifica) beds in different geographic settings in Southern California
Source: PLoS One. 2023 Oct 5;18(10):e0286228. doi: 10.1371/journal.pone.0286228 (PMC10553302; doi:10.1371/journal.pone.0286228)
Supplement: Attachment — Submitted filename: PONE-D-23-14325_review.pdf [file pone.0286228.s006.pdf]

This is an interesting paper looking at diversity in seagrass in embayment areas, mainland open coasts, and island open coasts, and identified distinct fish communities in each location using environmental DNA (eDNA). The findings emphasize the effectiveness of eDNA analysis in characterizing local biodiversity patterns. Additionally, the study demonstrated that eDNA analysis can closely replicate visual surveys while also detecting a wider range of marine fish species. This research is significant because it compares eDNA results with in-situ surveys, which is not commonly done in the literature and highlights the importance of conducting surveys in the exact sampling areas and approximately the same time as eDNA collection.

Therefore, this study is very important and very welcome. However, some major concerns prevent me from recommending the publication of this manuscript in its current form. Below, I have specified some major and minor issues which the authors could take into consideration to improve the overall quality of the manuscript.

The introduction, discussion, and literature cited of the study primarily focus on California, which overlooks the need for a broader context and comprehensive information on previous studies conducted on environmental DNA (eDNA) in seagrass meadows. These studies are highly relevant to the current research and should be adequately incorporated into the introduction to provide a more comprehensive understanding of the subject. Some relevant studies:

Angeles, I. B., Romero-Martínez, M. L., Cavaliere, M., Varrella, S., Francescangeli, F., Piredda, R., ... & Frontalini, F. (2023). Encapsulated in sediments: eDNA deciphers the ecosystem history of one of the most polluted European marine sites. *Environment International, 172*, 107738.

Qiu, S., Ooi, J. L. S., Chen, W., Poong, S. W., Zhang, H., He, W., ... & Loh, K. H. (2023). Heterogeneity of Fish Taxonomic and Functional Diversity Evaluated by eDNA and Gillnet along a Mangrove–Seagrass–Coral Reef Continuum. *Animals*, *13*(11), 1777.

Wesselmann, M., Geraldi, N. R., Marbà, N., Hendriks, I. E., Díaz-Rúa, R., & Duarte, C. M. (2022). eDNA Reveals the Associated Metazoan Diversity of Mediterranean Seagrass Sediments. *Diversity*, *14*(7), 549.

Stat, M., John, J., DiBattista, J. D., Newman, S. J., Bunce, M., & Harvey, E. S. (2019). Combined use of eDNA metabarcoding and video surveillance for the assessment of fish biodiversity. *Conservation Biology, 33*(1), 196-205.

Introduction: The current organization of the introduction requires significant reorganization, starting with a general context to specific research gaps. Additionally, there is a notable absence of relevant literature that should be included to provide a comprehensive understanding of the topic.

Line 60 – 63: Explain before how is the structural complexity of seagrass meadows (three dimensional habitat – from belowground components with network of roots and rhizomes to aboveground components) and which components are important for fish. Also, not sure what do you mean with unstructured habitats, be more specific. Do you mean bare sand? Other macrophytes?

Line 83-88: The paragraph is better placed in the earlier sections where the significance of seagrass meadows is discussed: they are are hotspots for biodiversity but have high regression rates and therefore it is necessary to monitor seagrass meadows continuously due to the changing ocean conditions and their already current regression rates.

Line 94: edna is not only used to follow biodiversity also to understand the diet of animals, trophic interactions…etc. include citation

Line 97: include citation

Line 104: Also include that visual census surveys are limited in scope as they tend to focus on specific taxa and are conducted at a local scale. Additionally, these surveys require taxonomic experts to accurately identify and classify organisms. Therefore, there is a potential for misidentification, which can introduce errors or uncertainties in the collected data.

Line 106: Include in this section what has been done in edna in seagrass meadows (both in water and sediments) in the world and in California.

Methods: To understand better the sampling design, Include a table with date of collection, site, location, seagrass meadow/bare sand, open coast/embayment, depth, geographic coordenates, visual survey conducted or not

Lien 143-149: What were the depths of the sampling sites? Same depth among sites?

Line 164: use citation number to be conssitent though the text

Line 403: this paragraph fits better at the end of the discussion

Figure 1: Heading missing

Figure 6: how can be that the number of species found in both surveys is bigger than the sum of edna and scuba survey?

Figure 4: Fit fish species on the ordination so that their relative length indicate the correlation between species and the NMDS.
